# Seizure recurrence after hemispheric disconnection—An EEG false lateralization trap

**DOI:** 10.1002/epd2.70253

**Published:** 2026-04-22

**Authors:** Prashant Natteru, Ajay Gupta, Majed Alzahrany, William Bingaman, Ahsan Moosa Naduvil Valappil

**Affiliations:** ^1^ Epilepsy Center, Neurological Institute, Cleveland Clinic Children’s Cleveland Ohio USA; ^2^ Department of Neurology Mayo Clinic Health System La Crosse Wisconsin USA; ^3^ Department of Neurology King Abdulaziz University Jeddah Saudi Arabia

Seizure recurrence after hemispheric disconnection requires careful evaluation to distinguish seizures due to incomplete disconnection in the operated hemisphere from independent contralateral hemisphere epileptogenicity.^1^, ^2^, ^3^, ^4^ Interpretation of scalp EEG in this postoperative setting poses unique challenges and may be misleading.^5^, ^6^ We present a clinical vignette of Rasmussen encephalitis demonstrating false lateralization on EEG after functional hemispherectomy, with seizure freedom achieved following conversion to anatomic hemispherectomy.

A 10‐year‐old right‐handed girl developed epilepsy at age 7 years. Initial seizures consisted of brief left facial and arm clonic jerking. Development and neurological examination were normal. EEG demonstrated right frontotemporal epileptiform discharges (Figure S1A), and MRI revealed focal atrophy around the right inferior precentral sulcus (Figure S2A). Over the next 3 years, seizure burden increased with emergence of atypical absences, drop attacks, and bilateral tonic–clonic seizures, occasionally preceded by left facial clonic activity. Interictal and ictal EEG showed generalized epileptiform discharges, at times preceded by frontocentral discharges (Figure S1B–D). Serial MRI brain demonstrated progressive right hemispheric atrophy (Figure S2B), consistent with Rasmussen encephalitis.^7^ Brain FDG‐PET showed concordant focal hypometabolism (Figure S2C). Autoimmune and genetic testing were unrevealing. Seizures remained refractory despite trails of several antiseizure medications and immunomodulatory therapy with intravenous immunoglobulin for 2 years. At age 11, the patient underwent a functional right hemispherectomy, as the family elected to accept the expected hemiplegia in hopes of seizure freedom. Histopathology showed chronic inflammatory infiltrates, microglial nodules, and gliosis consistent with Rasmussen encephalitis.

Four months after surgery, seizures recurred. Interictal EEG showed a higher burden of epileptiform discharges in the left parasagittal region (60–65%) than the right hemisphere (35–40%) (Figure 1A,B). Bilateral asymmetric tonic seizures, with flexion of the paretic left arm and extension of the right arm, were recorded. Ictal EEG showed robust left hemispheric patterns (Figure 1C). Several focal impaired consciousness seizures with left frontocentral ictal patterns were also recorded (Figure 1D). On magnetoencephalogram, interictal and ictal dipoles were localized to the left hemisphere (Figure S3A). Postoperative brain MRI, including diffusion tensor imaging, showed no residual connections (Figure S3B).

**FIGURE 1:**
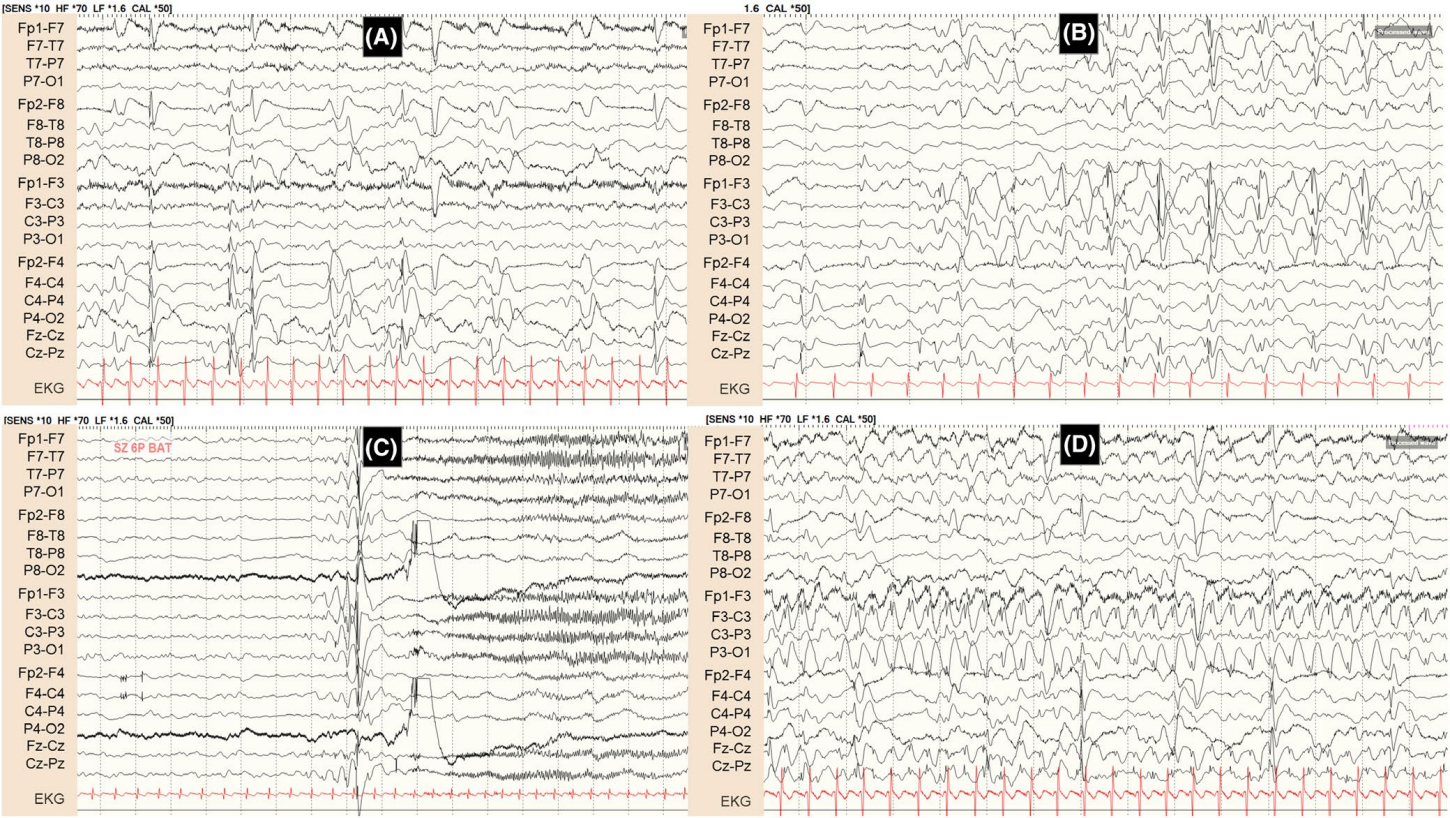
(A–D) EEG findings following seizure recurrence after functional hemispherectomy. (A) and (B): Interictal EEG demonstrates frequent multifocal sharp waves in the disconnected right hemisphere (A) and frontocentral spikes in the left hemisphere (B). (C): Ictal EEG showing lateralized low‐voltage fast activity in the left hemisphere during a bilateral asymmetric tonic seizure. (D) Additional ictal events characterized by discharges in the left frontocentral region, associated with either no clinical signs or a subtle behavioral pause.

Given persistent seizures and the known limitations of EEG lateralization after hemispheric disconnection, a right anatomic hemispherectomy was performed. The patient achieved sustained seizure freedom with resolution of contralateral epileptiform activity (Figure S4). Antiseizure medications were successfully discontinued gradually 1 year after surgery, and she has remained seizure‐free at the last follow up, 5 years after surgery.

Our case illustrates two critical interpretive pitfalls following functional hemispherectomy. First, ictal and interictal EEG lateralization to the contralateral hemisphere does not reliably indicate independent contralateral epileptogenicity. Second, absence of an overt residual connection on postoperative MRI does not exclude incomplete disconnection. Seizure freedom after anatomic hemispherectomy in our patient confirms that there was a residual connection between the hemispheres and the preoperative EEG findings were false lateralizing.

Following disconnective surgery, seizures arising from the operated hemisphere may generate insufficient synchronized cortical activity for scalp EEG detection until propagation to the contralateral hemisphere, potentially yielding misleading lateralization. The presence of abundant generalized abnormalities prior to functional hemispherectomy in our patient may be a factor for such paradoxical patterns after hemispheric disconnection. Similar false lateralization of scalp EEG in hemispherectomy candidates with extensive lesions with volume loss has been described.^5^ Conversely, interictal epileptiform discharges in the disconnected hemisphere are common and in seizure‐free patients these findings are clinically inconsequential.^6^

MRI remains essential for detecting residual connections, commonly involving the genu or splenium of the corpus callosum, temporal stem, insula, and frontobasal regions.^2^ Successful seizure freedom in our patient after anatomical hemispherectomy shows that the absence of residual connections on postoperative MRI should not be interpreted as definitive evidence of complete disconnection.

Reoperative hemispherectomy has been shown to result in seizure freedom in 20–33% of patients and substantial improvement in many others.^3^, ^4^, ^8^, ^9^ In one series of Rasmussen encephalitis, all five patients who needed reoperation after initial functional hemispherectomy achieved seizure freedom.^4^ Absence of structural abnormalities in the left hemisphere and rarity of true bilateral disease in Rasmussen encephalitis and high seizure burden in our patient prompted us to proceed with anatomic hemispherectomy.

This case underscores the need for cautious interpretation of postoperative EEG and MRI findings after functional hemispherectomy. Recurrent seizures after hemispheric disconnection warrant consideration of an incomplete disconnection even in the absence of supportive evidence on MRI and EEG.

## CONFLICT OF INTEREST STATEMENT

The authors declare no conflict of interest.

**Test Yourself**

1. A routine EEG done in an 8‐year‐old girl who has been seizure free for 2 years after hemispheric disconnection for Rasmussen encephalitis shows frequent repetitive epileptiform discharges in the operated hemisphere. Which of the following is the right next step?
  - Prolonged video EEG monitoring to capture seizures
  - Brain MRI to assess for incomplete disconnection
  - Increase antiseizure medications
  - No additional testing is needed
  - Consider immunotherapy
2. A 12‐year‐old boy began having recurrent seizures 4 months after right disconnective hemispherectomy for Rasmussen encephalitis. Video EEG showed ictal patterns maximum over the left hemisphere. Which of the following is the right next step?
  - Brain MRI to assess for incomplete disconnection
  - Increase antiseizure medications
  - Immunotherapy is warranted for bilateral Rasmussen disease
  - Perform PET study to assess the unoperated hemisphere
  - Stereo‐EEG to localize epilepsy from the left hemisphere
3. Which of the following is not a common feature of Rasmussen encephalitis?
  - Epileptia partialis continua
  - Progressive hemiparesis
  - Unilateral cortical atrophy
  - Chronic inflammation and microglial nodules on histopathology
  - Excellent response to immunomodulatory treatment

*Answers may be found in the* *Supporting Information*.

## Supporting information


**Figure S1.** (A–D). Interictal and ictal EEG findings before hemispheric disconnection. (A) Interictal EEG at age 7 years showing focal epileptiform discharges over the right fronto‐centro‐temporal regions (black arrow). (B) Interictal EEG at age 10 years demonstrating frequent bilateral frontocentral spikes and generalized spike–wave complexes. (C) and (D): Ictal EEG epochs recorded at age 10 years, illustrating distinct seizure types. Generalized ictal pattern with low amplitude fast activity associated with a generalized tonic seizure preceded by a myoclonic jerk (C). Note the bilateral parasagittal spikes preceding the seizure, resembling the interictal discharges.: Generalized spike discharges followed by brief attenuation, corresponding with whole‐body myoclonic jerks (D).

Data S1.

## Data Availability

The data that support the findings of this study are available from the corresponding author upon reasonable request.
