# Supplementary material for: Seizure recurrence after hemispheric disconnection—An EEG false lateralization trap
Source: Epileptic Disord. 2026 Apr 22;28(4):1309–12. doi: 10.1002/epd2.70253 (PMC13499243; doi:10.1002/epd2.70253)
Supplement: Supplementary file 1 [file EPD2-28-1309-s001.docx]

Answers

1. Answer is D. Epileptiform discharges, including lateralized periodic discharges and electrographic seizures without clinical signs or symptoms may be observed in the disconnected hemisphere even in seizure-free patients. In one study, 80% of patients who were seizure-free after hemispheric disconnection had ipsilateral epileptiform discharges. Therefore, no additional testing is warranted in this patient, and anti-seizure medications may be gradually weaned provided there is no evidence of independent epileptogenicity in the contralateral hemisphere.
2. Answer is A. In patients with seizure recurrence after disconnective hemispherectomy, prompt evaluation for incomplete disconnection is warranted. Video-EEG findings may be falsely lateralizing and should be interpreted with caution. Brain MRI remains the best available modality to identify residual interhemispheric connections, although it has inherent limitations. Bilateral Rasmussen encephalitis is exceedingly rare and should not be diagnosed without thorough evaluation to exclude incomplete disconnection. The role of stereo-EEG in this setting is uncertain and may be misleading, similar to scalp EEG findings. Escalation of antiseizure medications and immunotherapy may be considered only if true bilateral disease is confirmed.
3. Answer is E. Epilepsia partialis continua, progressive hemiparesis, unilateral cortical atrophy, and inflammatory changes with microglial nodules are characteristic features of Rasmussen encephalitis. The disorder is often poorly responsive to immunotherapy, and most patients do not experience sustained or meaningful improvement in seizure burden or disease progression.
